# Prenatal diagnosis and genetic analysis of a fetus with Branchio-oto-renal syndrome: A case report

**DOI:** 10.1097/MD.0000000000031172

**Published:** 2022-10-28

**Authors:** Ping Tang, Jiarui Li, Jun Li, Juan Yang, Jianjun Zhu

**Affiliations:** a Fetal Medical Center, Jiaxing Maternity and Children Health Care Hospital/The Affiliated Women and Children’s Hospital of Jiaxing University, Jiaxing, Zhejiang, China; b Department of Orthopaedic Surgery, The First Affiliated Hospital, Zhejiang University School of Medicine, Hangzhou, China; c Department of Surgery, Jiaxing Maternity and Children Health Care Hospital/The Affiliated Women and Children’s Hospital of Jiaxing University, Jiaxing, Zhejiang, China.

**Keywords:** Branchio-oto-renal syndrome, case report, EYA1 gene, genetic counseling, whole exome sequencing

## Abstract

Branchio-oto-renal (BOR) syndrome is an autosomal-dominant disorder characterized by branchial arch anomalies, hearing loss, and kidney defects. Mutations in the human EYA1 gene have been reported associated with BOR syndrome. Here we identified that a novel variant, EYA1: NM_000503.4: c.827-1G > C (Intron 8, shear mutation) was associated with BOR in a fetus of a Chinese family.

**Case presentation:**

Prenatal ultrasound examination showed that both kidneys of the fetus were small and the echo of both kidneys was enhanced. The amount of amniotic fluid was normal, and no other structural abnormalities of the fetus were found. Fetal umbilical cord blood puncture was performed. No abnormality was found in karyotyping and chromosomal microarray analysis (CMA) results. Thus, we performed a trio-based whole exome sequencing (WES), and found that the fetus carried a novel homozygous variant, EYA1: NM_000503.4: c.827-1G > C (Intron 8, shear mutation), but the parents do not have this mutation. The variation sites of fetus and parents were verified by Sanger sequencing to clarify the source of pathogenic variation.

**Conclusion:**

Combined with fetal imaging examination, the novel variation of EYA1: NM_000503.4: c.827-1G > C is the cause of fetal renal dysplasia. This case report indicates that the early use of appropriate technology can clarify the etiology of fetal disease and guide prognosis consultation.

## 1. Introduction

Branchio-oto-renal (BOR) syndrome (OMIM#113650) is a rare autosomal dominant genetic disorder characterized by sensory neurologic, conductive, or mixed hearing loss, structural defects of the outer, middle, and inner ears, branchial slits, fistulas, or cysts.^[1,2]^ And renal abnormalities ranging from mild hypoplasia to complete absence.^[3]^ It is reported that the prevalence of BOR syndrome is approximately 1:40,000, of which approximately 2% are children with severe hearing loss with poor prognosis of bilateral renal dysplasia.^[4]^

The syndrome is clinically and genetically heterogeneous in the population, with high penetrance and a variety of manifestations, but only fetal renal dysplasia may be detected by imaging. It is reported that the BOR related pathogenic gene was located on chromosome 8q13.3 using linkage studies.^[5]^ Then, a novel causative gene, named as EYA1 was identified though several patients with BOR syndrome.^[6]^ EYA1 is a conserved transcriptional co-activator functions as a protein phosphatase, and is highly expressed in embryonic kidney of human. There are a number of clinical BOR patient cases,^[7–10]^ this case report is the first intrauterine diagnosis of BOR syndrome by whole exome sequencing and this sequence variant has not been previously reported.

## 2. Patients and Methods

### 2.1. Case presentation

**Figure 1.**
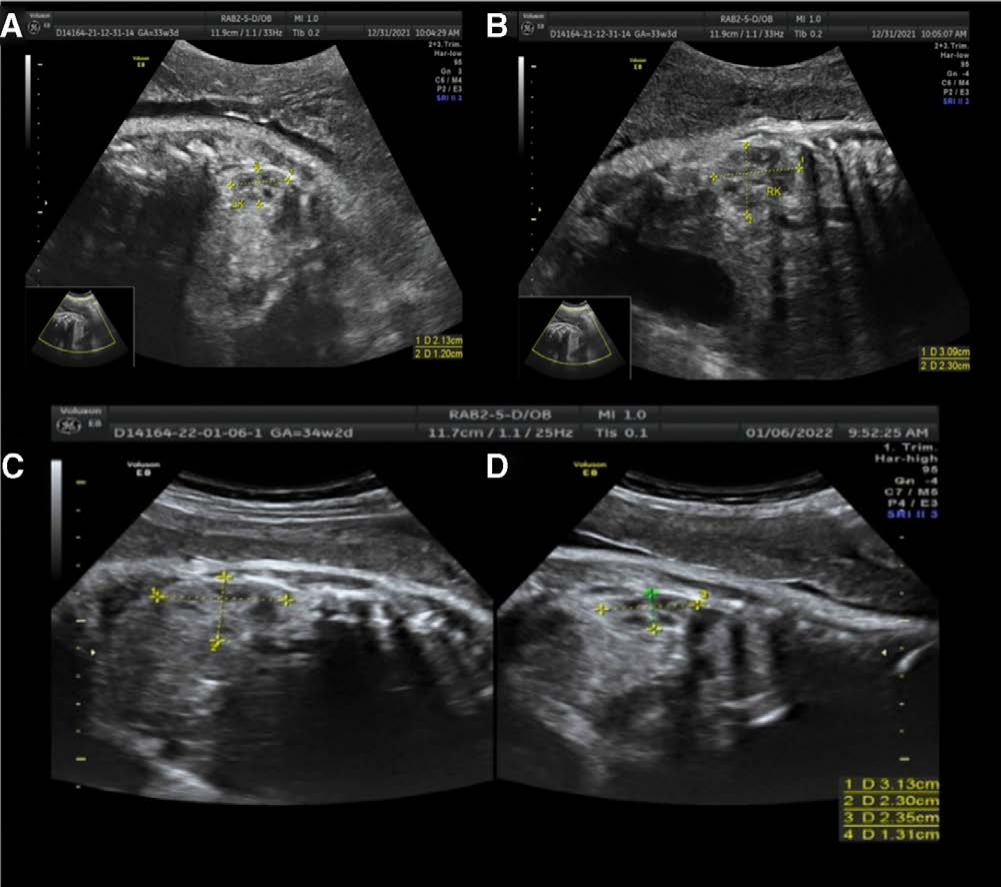
Ultrasound image of fetal kidney. A, B: Left and right kidney size at 33 weeks + 4 days; renal volume decreased and echo increased; C, D: 36 weeks + 3 days, the volume of both kidneys decreased and the echo increased.

A 28-year-old pregnant woman, was in the first pregnancy, has not delivered and was in good health. She denied a history of familial or genetic disease. Routine obstetric care was performed during pregnancy. Serological prenatal screening showed low risk and no abnormalities in prenatal ultrasound (double kidney size between –1 SD). In the pregnant metaphase, 2 ultrasounds showed that good fetal heart and fetal movement, but did not indicate the size of both kidneys. At the 33 + 4 weeks of pregnancy, ultrasound examination showed that the size of the left kidney was 21*12 mm (–5.09 SD, average longitudinal diameter is 44.72 mm), the anteroposternal diameter of the left renal pelvis was 6.7 mm, and the size of the right kidney was 31*23 mm (–2.94 SD) (Fig. 1A, B). The echo of both kidneys was slightly stronger, and the amniotic fluid volume was normal. Ultrasound examination of adult urinary system of fetal parents showed no abnormality.

### 2.2. Sample collection

Routine fetal cord blood punctures were performed to collect 3 mL fetal cord blood samples, and 5 mL venous blood samples from parents respectively. Informed consent was obtained from the parents, and the study was reviewed by Prenatal Diagnosis Ethics Committee of Jiaxing Maternal and Child Health Care Hospital (2019 PS 493 K).

### 2.3. Examination

After genetic counseling, pregnant women and their families voluntarily chose cord blood genetic testing (chromosome karyotype analysis, chromosomal microarray analysis, and trio-based whole exome sequencing). Chromosome karyotype and SNP-array analysis showed no abnormality. Thus, we performed trio-based whole exome sequencing to detect presence of any disease-related mutations, 2 mL of blood in ethylenediamine tetraacetic acid (EDTA) coated tube was sent to the ShenZhen BGI Medical Test Laboratory. The sequencing was performed using capture high-throughput chip technology, detection of almost 20,000 genes in the human genome. Sanger sequence were used to verify the mutations.

### 2.4. Whole exome sequencing

Deoxyribonucleic acid (DNA) extraction kit (Tiangen Biochemical Technology Co., LTD.) was used to extract DNA from umbilical cord blood and peripheral blood from the parents for purity and quantitative detection, and the remaining samples were stored at –20 ℃ for future use. Polymerase chain reaction (PCR) amplification products were purified and sequenced by Shenzhen BGI Co., LTD. Candidate mutation sites were obtained after DNA extraction and quality inspection, genome library construction, whole exome sequencing (WES), Sanger sequence, and bioinformation analysis.

### 2.5. Sanger sequence validation

According to the results of WES, sanger sequencing was used to validate the mutation sites of the fetus and parents. Corresponding PCR primers were designed for the suspected pathogenic sites, PCR amplification and product purification were performed, and the sequencing results were sequenced on ABI3130. BLAST software was used to compare the sequencing results with the standard sequence of GenBank to determine the mutation sites. The pathogenicity of mutated loci was judged according to the classification criteria for genetic variation formulated by the American College of Medical Genetics and Genomics (ACMG) in 2015. The classification of variation pathogenicity is based on the sequence variation Interpretation guidelines of the ACMG and the American Society for Molecular Pathology (AMP), and the detailed interpretation of the guidelines is based on the ClinGen Sequence Variation Interpretation Working Group and the British Society for Clinical Genomic Sciences (ACGS).

## 3. Results

After 3 weeks, the ultrasound amniotic fluid index was 133 mm, the left kidney size of the fetus was 24*13 mm (–4.65 SD), and the size of the right kidney was 31*23 mm (–3.17 SD). The echo of both kidneys was slightly stronger, the bilateral renal arteries and the bladder were visible (Fig. 1C, D).

**Figure 2.**
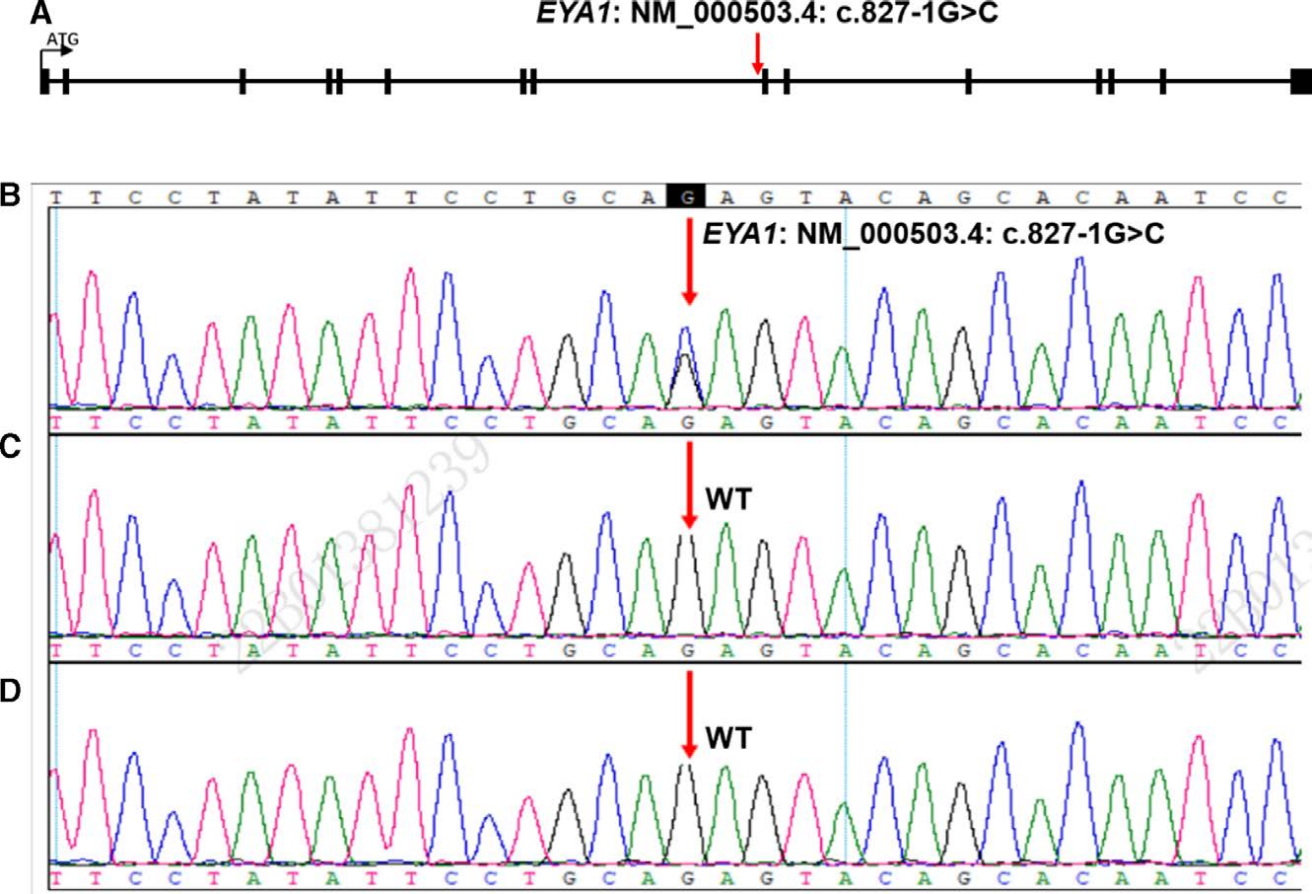
The results were verified by fetal Sanger sequencing. A: The position of mutation, NM_000503.4: c.827-1G > C on EYA1. B: EYA1: c.827-1G > C in fetus. C: NM_000500.4 (EYA1): C.827-1G in fetal mother is wild homozygous type. D: NM_000500.4 (EYA1): C.827-1G fetal father is wild homozygous type.

The results showed that one suspected pathogenic mutation was located on chromosome 8; chr8: 72184133, detected in EYA1 gene, which was partially related to the phenotype of the BOR syndrome. The fetus carried the novel homozygous variant, EYA1: NM_000503.4: c.827-1G > C, but the parents do not have this mutation (Fig. 2).

According to the results of genetic testing, the possible prognosis of the fetus was consulted in detail. The pregnant woman and her family requested to abandon the fetus, and the hospital ethics discussion and approval were performed, and the childbirth was induced.

## 4. Discussion

BOR is currently diagnosed in symptomatic individuals, but no prenatal diagnosis has been reported. Phenotypic heterogeneity of BOR is strong, which can cause extremely severe deafness and complete loss of renal function.^[11,12]^ Prenatal diagnosis can help families understand fetal prognosis and make prudent decisions about pregnancy. Due to the clinical heterogeneity and molecular genetic characteristics of BOR, there are currently no diagnostic criteria for prenatal fetal BOR.^[13]^ Exploring the intrauterine symptoms of fetal BOR and further clarifying the pathogenic genes and their mutations can obtain an accurate etiology, which is helpful for further Genetic counseling.

Due to amniotic fluid volume, ultrasound examination only suggested that the fetal double kidneys were small with enhanced echo, making it difficult for clinical consultation to accurately judge the prognosis. Genetic identification of the pathogenic molecular causes of this phenotype is of significance for prognosis judgment. Ultrasound examination is the preferred imaging examination method to diagnose fetal urinary system abnormalities.^[14,15]^ Using a high-resolution ultrasound probe to explore the abdomen, the fetal kidney can be displayed at 14 weeks, and be constantly displayed at 18 weeks. When the fetal urinary system is malformed, the size of the kidney can be intuitively shown. For example, when the kidney is dysplasia, the size of the kidney is reduced or even disappeared. In this case, the fetal kidney was obviously small at 32 weeks of pregnancy, which attracted our attention. With the increase of gestational age, the fetal kidney development was not synchronized with the increase of gestational age, and there was no decrease in amniotic fluid, which caused difficulties in prognosis judgment.

Dynamic monitoring of kidney size is particularly important for prenatal diagnosis of renal dysplasia.^[16,17]^ There are many mechanisms leading to congenital urinary system abnormalities, among which chromosome abnormality and gene mutation are the most important causes.^[18]^ Some BOR pathogenic genes, including EYA1 (8q13.3), SIX1 (14q23.1), and SIX5 (19q13.32) have been identified.^[6,19,20]^ Among them, EYA1 is the first identified and the most common pathogenic gene that causes BOR.^[6]^ Nearly about 40% of BOR patients have EYA1 mutations. As of January 2021, the HGMD database (www.hgmd.cf.ac.uk/ac/index.php) has collected 240 EYA1 mutations associated with BOR. There are several BOR cases reports in Chinese population, and only a total of 14 pathogenic/suspected pathogenic mutations of EYA1 in Chinese population have been recorded in database and literature.^[21–25]^ In this study, the heterozygous mutation of EYA1: NM_000503.4: c.827-1G > C was first found in Chinese population. We identified the pathogenic gene associated with normal amniotic fluid and reduced fetal double kidney shape through trio-based whole exome sequencing, providing better prognostic advice for the fetus.

It is often difficult to make prenatal diagnosis of some fetal syndromes due to the lack of clinical phenotypes. Counseling informs the prognosis of the gene mutation, which may cause postnatal renal dysplasia, the possibility of severe deafness, and other genealogical symptoms. Although the fetus has a novel mutation, prenatal diagnosis is still required for another pregnancy to rule out the risk of gonadal mosaicism, which plays an important role in clarifying disease diagnosis and guiding fertility. With the comprehensive consideration of karyotype analysis, chromosomal microarray analysis, whole exome sequencing, and combined with the serious urinary system malformations, such as polycystic kidney, the prognosis of the fetus is extremely poor, and it is recommended to induce labor. If the presence of amniotic fluid on continuous ultrasound monitoring is minimal, it indicates that fetal renal function is severely impaired.

## Author contributions

JZ designed the study and reviewed the manuscript. PT and RJL analyzed the data and wrote the manuscript. PT, JL and JY performed the experiments. PT and RJL collected the data. All authors have read and approved the manuscript.

**Conceptualization:** Jianjun Zhu.

**Data curation:** Ping Tang, Jiarui Li, Juan Yang.

**Formal analysis:** Jun Li.

**Funding acquisition:** Ping Tang.

**Investigation:** Jiarui Li.

**Methodology:** Jun Li, Juan Yang.

**Project administration:** Jianjun Zhu.

**Writing – original draft:** Ping Tang, Jiarui Li.

**Writing – review & editing:** Jianjun Zhu.
